# Recurrent Pelvic Organ Prolapse after Sacrocolpopexy—A Surgical Challenge

**DOI:** 10.3390/jcm13061613

**Published:** 2024-03-12

**Authors:** Andreas Martin Studer, Ivo Faehnle-Schiegg, Janine Frey, Simone Aichner, Christine Brambs, Corina Christmann-Schmid

**Affiliations:** Department of Urogynecology, Cantonal Hospital of Lucerne, Spitalstrasse, 6000 Lucerne, Switzerland

**Keywords:** recurrent POP, pelvic organ prolapse, repeat sacrocolpopexy, laparoscopic sacrocolpopexy, sacrocolpopexy

## Abstract

**Background**: Repeat sacrocolpopexy (reSCP) for recurrent pelvic organ prolapse (POP) is a rare and complex condition with little understanding of how to manage. Most authors recommend complete reSCP regardless of the underlying cause of the failure. This retrospective cohort study presents our management workflow and how to systematically approach this challenging situation. **Methods**: From 2017 to 2021, we analyzed all women undergoing surgery for recurrent POP after sacrocolpopexy at our tertiary referral hospital at the department of urogynecology. Preoperatively, all women underwent a structured work-up consisting of answering the validated German female pelvic floor questionnaires, a clinical examination utilizing the POP-Q staging system according to the International Continence Society (ICS), and a pelvic floor ultrasound. The surgical management was based on the preoperative findings and was adapted individually during surgery if indicated according to the estimated underlying problem for recurrence. **Results**: In total, 377 women underwent a primary laparoscopic sacrocolpopexy. However, ten women presented with a symptomatic recurrent prolapse requiring further surgical intervention. A reSCP was performed in eight women, including two with additional laparoscopic paravaginal repair to correct the displaced mesh placement at initial surgery. A vaginal correction was indicated in two women with an isolated posterior compartment prolapse. The analysis demonstrates that reSCP has a low intraoperative complication rate and high subjective and objective success rates. **Conclusions**: We could demonstrate that individualized reSCP after initial SCP is a challenging yet feasible and safe treatment option, but there may be suitable alternatives. If women undergo pre- and intraoperative standardized problem-oriented examinations, we can often identify the cause of the recurrent prolapse. Tailored surgery must be subsequently performed.

## 1. Introduction

Sacrocolpopexy (SCP) is the gold standard treatment option for the surgical management of apical and multicompartment pelvic organ prolapse (POP) [1,2], with high subjective and objective success rates in the short and long term [3,4,5,6,7]. Despite the low recurrence rate, there are women presenting with bothering recurrent POP after SCP, requiring further surgical intervention [8,9,10]. More urogynecologists will be asked to manage this challenging situation since future prolapse surgery is not only performed in older women and patients want to preserve their quality of life up until old age [11]. After searching the PubMed and Cochrane libraries, we discovered that there are no systematic reviews on how to systematically approach recurrent POP surgically after failed SCP. The limited data [12,13,14,15] mostly recommend a complete repeat sacrocolpopexy, assuming the redo procedure tackles the primary shortfalls and weaknesses. Disregarding the few weaknesses of sacrocolpopexy to correct concomitant pathologies without severe complication risks. Therefore, we presented our decision process based on the outcome data of primary cases, supposing equivalent performance in repeat SCP.

Minimal invasive treatment options for repeat apical and multicompartment prolapse are limited. Outcome data on reSCP are also limited. Alternative surgical intervention techniques such as pectopexy [16,17,18,19] or lateral suspension [20,21] do not treat all compartments and have inferior long-term data compared to SCP [17,19,21,22]. Vaginal treatment options, including vaginal native tissue repair, correcting the anterior [23] and/or apical compartment [24], have lower long-term success rates compared to the abdominal approach [22,25]. Due to the FDA warning in July 2011 [26], further options, such as transvaginal meshes, are not widely available due to the high complication rate [27]. The primary aim of this retrospective study was to elaborate a systematic and standardized pre- and intraoperative work-up in women with recurrent POP after SCP. Secondly, we aimed to demonstrate that repeat SCP is a challenging yet feasible and safe treatment option in well-selected patients with high subjective and objective outcomes, assuming an insufficient primary surgery as a reason for recurrent prolapse. Alternatives such as laparoscopic paravaginal repair or posterior colporrhaphy are appropriate if a defect seems to be arising from a composite compartment.

## 2. Materials and Methods

All women undergoing POP-related surgery after SCP from January 2017 to December 2021 were included in this retrospective cohort study at our tertiary referral hospital in Switzerland.

Recurrent POP was defined as POP-Q point D ≥ −2 and/ or Ba/Bp ≥ 0. A comprehensive chart review was completed, including the patients’ previous history and urogynecology interventions in the past. To objectify the clinical examination, the POP-Q quantification system according to the ICS [28] was used pre- and 6–8 weeks postoperatively. In all women, a perineal and transvaginal ultrasound [29,30] was performed to assess the pelvic floor and the placement of the mesh anteriorly, posteriorly, and at the level of the apex. The findings were noted separately and taken into consideration regarding the surgical management of recurrent POP. To evaluate the subjective success, women completed the validated German female pelvic floor questionnaire pre- and postoperatively [31]. The questionnaire covered the domains of bladder, bowel, prolapse, and sexual dysfunction. The higher the score, the worse the symptoms. 

**Figure 1:**
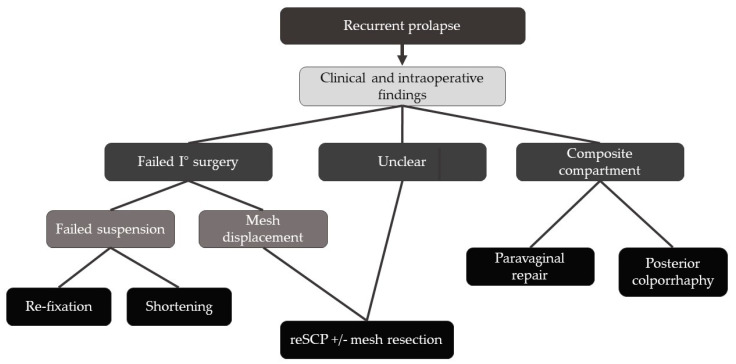
Simplified management workflow.

Based on the suspected underlying cause of the recurrent POP and intraoperative findings concerning the anterior or posterior compartment as well as the suspension, we adapted our approach as summarized in Figure 1.

After estimating the cause for recurrence, we tailored our consultation regarding the failed primary surgery or lacking composite compartment consenting for intraoperative adaptation according to its findings.

The intraoperatively structured evaluation of the pelvis was performed via the following steps:

1. The examination of the integrity of the suspension arm:
  - A too-long or floppy suspension of the Y-shaped mesh with sufficient anchorage at the level of the sacral promontory. In this scenario, the shortening of the suspending mesh using non-resorbable sutures was performed;
  - Detachment or insufficient anchorage at the level of promontory re-fixation or substitution is applicable.
2. Concomitant examination of the mesh placement anteriorly, apically, and posteriorly:
  - The dissection of the anterior vesicovaginal and/or posterior rectovaginal space. In the case of an underlying failure at the level of the vagina, a complete or partial SCP, according to Christmann-Schmid et al. [32], was performed using an EndoGYNious polypropylene mesh produced by A.M.I.^®^, Feldkirch, Austria;
  - A mesh excision or overlay was based on the intraoperative findings and patients’ preoperative symptoms.

The limitation of the above procedure is that the right pelvic sidewall does not have enough remaining peritoneum for the subsequent peritoneal closure of the new mesh.

3. Non-treated defects:
  - In the case of a symptomatic low rectocele but all the other compartments are well-suspended, an isolated posterior colporrhaphy [33,34] was performed;
  - A lateral defect of the anterior compartment not treated with the initial SCP was surgically addressed with a laparoscopic paravaginal repair [35,36,37].

Composite compartment defects were treated concomitantly with reSCP or as a two-step approach if indicated. 

Perioperative complications regarding viscus injury, mesh extrusion, blood loss, and prolonged operation time were noted separately.

Due to the descriptive outline of this study, the statistical analysis was not constructive.

The study was approved by the local ethics committee (EKNZ 2021-00718, date of issue 11 May 2021).

## 3. Results

Out of the 548 patients with prolapse surgery, 377 received a SCP in the analyzed period, and only 10 women were identified with recurrency after sacrocolpopexy and included in this analysis. A total of 70% (7/10) of the women with recurrent POP after SCP had the initial surgery at our department. They all had undergone a SCP described in Christmann-Schmid et al. [32]. The remaining three women had their previous surgery at another institution in Switzerland. As extracted from the operative reports in these cases, the polypropylene mesh was only placed on the apex or at the apical posterior wall of the vagina and not to the level of the bladder neck or posteriorly down to the level of the perineal body. 

An adjusted repeat SCP was performed in eight women and singular combined with laparoscopic paravaginal repair. Two women received an isolated posterior repair. The median age of our patients was 61.5 years, and the time to recurrence varied from immediately after surgery to nine years. The operation time of the individualized repeat SCP, including additional interventions, varied from 80 to 192 min and averaged 128 min. Complications or adverse events emerged in three cases, with one postoperative mesh exposure and two bladder injuries, including one cystotomy due to major scarring. Blood loss was consistently below 20 mL. The demographics and operational details are summarized in Table 1.

Table 2 summarizes the underlying cause identified intraoperatively as well as the resulting surgical approach. A torn or loose mesh at the level of the sacral promontory was identified in 5/10 cases (cases 2, 4, 5, 7, and 8). A dislocated mesh anteriorly and/or vaginal vault was intraoperatively found in five women (cases 3, 4, 5, 6, and 7). An additional paravaginal defect or a floppy fixation was found in two women.

Additional continence surgery or mesh-related issues such as mesh extrusion was addressed secondarily whereas paravaginal repair and posterior colporrhaphy were performed once each concomitantly with repeat SCP. 

The subjective outcome findings are shown in Table 3. Preoperatively, all women reported bulge symptoms (question 28) [31], and seven had bladder issues, consisting of five having stress urinary incontinence (SUI), four had increased urgency, and one had incomplete bladder emptying. Two women with a remaining low rectocele after primary SCP reported stool outlet symptoms in the questionnaire, and three women stated sexual discomfort. 

A high subjective success rate was achieved. Postoperatively, two women reported persisting SUI and one symptomatic persistent rectocele.

A postoperatively high objective success rate was seen in most women. However, one woman had persisting POP in the posterior compartment and declined posterior colporrhaphy after repeat SCP, which is presented in Table 4.

## 4. Discussion

This retrospective cohort study demonstrated that repeat SCP is a feasible and safe surgical treatment option in well-selected and preoperatively clinically assessed women. 

Due to the small sample size, the demographics and complication rate were difficult to interpret but were in the expected range and comparable to the few published reports [13,15], with adequate operation times and blood loss similar to our cohort [32]. The size of the collective was certainly one of the biggest limitations of this study. However, it also highlighted the difficulty of investigating rare conditions, considering only roughly 2% of surgeries are repeat SCP.

Agreeably, primary failed surgery or untreated defects led to a short interval of the appearance of relapses, whereas meshes that were not placed low enough and weaknesses in the lateral compartment developed recurrency over a longer time.

Considering the intraoperative findings leading to recurrence, this data supports our approach of primarily taking an individualized repeat SCP into account, as displaced mesh could be demonstrated in seven out of ten women. Therefore, we decided to repeat SCP in two cases (5,6), as it was suspected that displaced/torn mesh occurred even if sufficient apical support was apparent. In isolated posterior de Lancey level III, recurrency or additional lateral defects, we achieved advantageous results via either posterior colporrhaphy or laparoscopic paravaginal repair due to specifically targeting the insufficient compartment.

Overall, the performance results demonstrate a satisfactory outcome not only in anatomical correction but also due to the dramatically reduced symptoms with an expectable rate of stress urinary incontinence (SUI) of 20% and only one case with minor bulging symptoms because of persisting rectocele (case six). 

After reviewing the limited published data, we consider an individualized repeat SCP as a preceding option in the instance of failed or inadequate primary sacrocolpopexy. Repeat SCP is a challenging but feasible and safe surgical option. To address incorrectly placed mesh or torn fixation, a re-do of the intervention can be successful as the primary procedure is regarded as the gold standard treatment to correct apical and multicompartment prolapse in comparison to the lower long-term objective and subjective success rates of native tissue procedures [22].

Furthermore, the short-term results of the RCTs comparing SCP versus vaginal mesh repair in women with an anterior and apical compartment prolapse demonstrated higher objective success rates in the SCP group [38,39].

Alternative laparoscopic surgical techniques, such as pectopexy [16,17,18,19] or lateral suspension [20,21], do not treat all compartments and do not have higher objective and subjective long-term success rates compared to SCP [17,19,21,22].

One limitation of the study is the potential impact of reSCP on bladder or bowel function could not be determined in this small cohort. Presumably, reSCP has a stronger negative impact than in primary surgery as the dissection plans to preserve the hypogastric nerve are challenging. Additional preparation at the promontory site may lead to increased constipation rates. Although, this was not reported in comparable studies [12,13,14,15].

Alternative mesh procedures such as pectopexy or lateral suspension may have a similar risk for bladder dysfunction due to equivalent anterior dissection. However, data are missing.

We consider the modification of reSCP utilizing the existing mesh or a reduction to fewer compartments feasible when sufficient components are apparent.

Generally, the two most limiting factors for a repeat SCP are the inability of the retroperitoneal closure of the subsequent SCP on the right pelvic sidewall due to extensive scarring and sacral promontory obstruction from the initial mesh fixation. However, neither of these factors was apparent in our case series. In the situation of impossibility for reSCP, laparoscopic alternatives like pectopexy [40] or lateral suspension may be applied.

## 5. Conclusions

In the event of disturbing prolapse recurrence after sacrocolpopexy, we recommend appropriate clinical and sonographic methods as well as an intraoperative assessment to determine the cause of failure. Once the cause is identified, it should be addressed specifically by either a repeat complete or partial sacrocolpopexy if the primary surgery is executed insufficiently. Alternatively, one should correct the isolated compartments via laparoscopic paravaginal repair or posterior colporrhaphy (Figure 1).

## Figures and Tables

**Table 1:** Baseline demographics including age = age at recurrency surgery (in years) and TTR = time to recurrency (in years).

Case	Age	TTR	BMI	Operation Time	Blood Loss	Complications	POP Surgery Following reSCP
Repeat sacrocolpopexies							
1	53	1.4	27	96	<20 mL	-	Anterior colporrhaphy *, TVT-O
2	70	0.6	27.3	90	<20 mL	-	Paravaginal repair
3	64	7.1	29.3	122	<20 mL	-	Posterior colporrhaphy
4	70	9.1	24.4	80	<20 mL	Mesh exposure	Partial mesh excision
5	59	0.3	25	150	<20 mL	Cystostomy	Anterior and posterior colporrhaphy, Sacrospinous fixation *, TVT
6	64	4.1	29.5	165	<20 mL	-	-
7	56	6.6	31.6	192	<20 mL	Bladder perforation	-
8	47	6.0	20.3	132	<20 mL	-	-
Posterior colporrhaphy							
9	70	0.4	25.2	22	<20 mL	-	-
10	52	0.3	24.2	35	<20 mL	-	-

* Recommended paravaginal repair.

**Table 2:** Intraoperative findings and surgical details for repeat sacrocolpopexies.

Case	Underlying Problem	Additional Surgery	Mesh Action	
Repeat Sacrocolpopexies		(during reSCP)	Existing Mesh	New Mesh
1	Unclear, secondary lateral defect	Posterior colporrhaphy	Partial resection	Re-use of existing anterior mesh *
2	Torn suspension, secondary lateral defect	-	Left in place	New mesh over existing mesh
3	Mesh only on the apex	Salpingectomy	Left in place	New mesh over existing mesh
4	Mesh only posterior and teared suspension	Adenectomy	Left in place	New mesh over existing mesh
5	Anterior mesh is not low enough, too-floppy suspension	-	Left in place, shortening of suspension	New anterior mesh
6	Insufficient mesh placement (rotated)	-	Complete resection	New mesh
7	Anterior mesh is not low enough, torn suspension	-	Left in place	New mesh over existing mesh
8	Too-floppy suspension, lateral defect	Paravaginal repair	Left in place, shortening of suspension	No new mesh

* No anterior preparation (major scarring).

**Table 3:** Pre- and postoperative symptoms (summary of the GFPF questionnaire).

Symptoms	Preoperative	Postoperative
Bladder function	7/10	2/10 *
Bowel function	2/10	0/10
Pelvic organ prolapse	10/10	1/10 °
Impaired sexual function	3/10	0/10

* Stress urinary incontinence, ° Reduced symptoms.

**Table 4:** Pre- and postoperative POP-Q measures.

**reSCP**	**POP-Q**					
	Preoperative			Postoperative		
	Aa	Ap	C	Aa	Ap	C
Mean	0	−2	0	−3	−3	−8
SD	1.1	1.1	3.8	0.8	1.1	1.5
**Colporrhaphy**	**POP-Q**					
	Preoperative			Postoperative		
	Aa	Ap	D	Aa	Ap	D
Mean	−3	−1	−13	−3	−3	−13
SD	0	0.5	0.5	0	0	0.5

## Data Availability

The raw data are available upon request from the corresponding author.

## References

[1] Costantini E., Brubaker L., Cervigni M., Matthews C.A., O’Reilly B.A., Rizk D., Giannitsas K., Maher C.F. (2016). Sacrocolpopexy for pelvic organ prolapse: Evidence-based review and recommendations. Eur. J. Obstet. Gynecol. Reprod. Biol..

[2] Maher C., Feiner B., Baessler K., Christmann-Schmid C., Haya N., Brown J. (2016). Surgery for women with apical vaginal prolapse. Cochrane Database Syst. Rev..

[3] Dwyer L., Kumakech W., Ward K., Reid F., Smith A. (2019). Laparoscopic sacrocolpopexy (LSCP) using an ultra-lightweight polypropylene mesh. Eur. J. Obstet. Gynecol. Reprod. Biol. X.

[4] Giugale L.E., Hansbarger M.M., Askew A.L., Visco A.G., Shepherd J.P., Bradley M.S. (2021). Assessing pelvic organ prolapse recurrence after minimally invasive sacrocolpopexy: Does mesh weight matter?. Int. Urogynecol. J..

[5] Thomas T.N., Davidson E.R.W., Lampert E.J., Paraiso M.F.R., Ferrando C.A. (2020). Long-term pelvic organ prolapse recurrence and mesh exposure following sacrocolpopexy. Int. Urogynecol. J..

[6] Vandendriessche D., Sussfeld J., Giraudet G., Lucot J.P., Behal H., Cosson M. (2017). Complications and reoperations after laparoscopic sacrocolpopexy with a mean follow-up of 4 years. Int. Urogynecol. J..

[7] Sarlos D., Brandner S., Kots L., Gygax N., Schaer G. (2008). Laparoscopic sacrocolpopexy for uterine and post-hysterectomy prolapse: Anatomical results, quality of life and perioperative outcome-a prospective study with 101 cases. Int. Urogynecol. J. Pelvic Floor Dysfunct..

[8] Hudson C.O., Northington G.M., Lyles R.H., Karp D.R. (2014). Outcomes of robotic sacrocolpopexy: A systematic review and meta-analysis. Female Pelvic Med. Reconstr. Surg..

[9] Pacquée S., Nawapun K., Claerhout F., Werbrouck E., Veldman J., D’hoore A., Wyndaele J., Verguts J., De Ridder D., Deprest J. (2019). Long-Term Assessment of a Prospective Cohort of Patients Undergoing Laparoscopic Sacrocolpopexy. Obstet. Gynecol..

[10] Sarlos D., Kots L., Ryu G., Schaer G. (2014). Long-term follow-up of laparoscopic sacrocolpopexy. Int. Urogynecol. J..

[11] Dieter A.A., Wilkins M.F., Wu J.M. (2015). Epidemiological trends and future care needs for pelvic floor disorders. Curr. Opin. Obstet. Gynecol..

[12] Grinstein E., Gluck O., Veit-Rubin N., Deval B. (2020). Laparoscopic management of pelvic organ prolapse recurrence after open sacrocervicopexy. Int. Urogynecol. J..

[13] Haya N., Maher M., Ballard E. (2015). Surgical management of recurrent upper vaginal prolapse following sacral colpopexy. Int. Urogynecol. J..

[14] Panico G., Campagna G., Vacca L., Caramazza D., Mastrovito S., Scambia G., Ercoli A. (2022). Redo laparoscopic sacrocolpopexy for POP recurrence: Is it the right call?. Eur. J. Obstet. Gynecol. Reprod. Biol..

[15] Ruess E., Roovers J.P., Jeffery S. (2020). Management of recurrent pelvic organ prolapse after sacrocolpopexy. A video case series. Int. Urogynecol. J..

[16] Najib B., Rusavy Z., Abdallah W., Deval B. (2023). Laparoscopic sacrocolpopexy in the management of recurrent pelvic organ prolapse. J. Gynecol. Obstet. Hum. Reprod..

[17] Noé K.G., Schiermeier S., Alkatout I., Anapolski M. (2015). Laparoscopic pectopexy: A prospective, randomized, comparative clinical trial of standard laparoscopic sacral colpocervicopexy with the new laparoscopic pectopexy-postoperative results and intermediate-term follow-up in a pilot study. J. Endourol..

[18] Szymczak P., Grzybowska M.E., Sawicki S., Futyma K., Wydra D.G. (2022). Perioperative and Long-Term Anatomical and Subjective Outcomes of Laparoscopic Pectopexy and Sacrospinous Ligament Suspension for POP-Q Stages II-IV Apical Prolapse. J. Clin. Med..

[19] Yang Y., Li Z., Si K., Dai Q., Qiao Y., Li D., Zhang L., Wu F., He J., Wu G. (2023). Effectiveness of Laparoscopic Pectopexy for Pelvic Organ Prolapse Compared with Laparoscopic Sacrocolpopexy. J. Minim. Invasive Gynecol..

[20] Dällenbach P. (2022). Laparoscopic Lateral Suspension (LLS) for the Treatment of Apical Prolapse: A New Gold Standard?. Front. Surg..

[21] Isenlik B.S., Aksoy O., Erol O., Mulayim B. (2023). Comparison of laparoscopic lateral suspension and laparoscopic sacrocolpopexy with concurrent total laparoscopic hysterectomy for the treatment of pelvic organ prolapse: A randomized controlled clinical trial. Int. Urogynecol. J..

[22] Kotani Y., Murakamsi K., Kai S., Yahata T., Kanto A., Matsumura N. (2021). Comparison of Surgical Results and Postoperative Recurrence Rates by Laparoscopic Sacrocolpopexy with Other Surgical Procedures for Managing Pelvic Organ Prolapse. Gynecol. Minim. Invasive Ther..

[23] Slade E., Daly C., Mavranezouli I., Dias S., Kearney R., Hasler E., Carter P., Mahoney C., Macbeth F., Delgado Nunes V. (2020). Primary surgical management of anterior pelvic organ prolapse: A systematic review, network meta-analysis and cost-effectiveness analysis. BJOG.

[24] Larouche M., Belzile E., Geoffrion R. (2021). Surgical Management of Symptomatic Apical Pelvic Organ Prolapse: A Systematic Review and Meta-analysis. Obstet. Gynecol..

[25] Lallemant M., Tresch C., Puyraveau M., Delplanque S., Cosson M., Ramanah R. (2021). Evaluating the morbidity and long-term efficacy of laparoscopic sacrocolpopexy with and without robotic assistance for pelvic organ prolapse. J. Robot. Surg..

[26] Urogynecologic Surgical Mesh: Update on the Safety and Effectiveness of Transvaginal Placement for Pelvic Organ Prolapse. https://www.fda.gov/files/medical%20devices/published/Urogynecologic-Surgical-Mesh--Update-on-the-Safety-and-Effectiveness-of-Transvaginal-Placement-for-Pelvic-Organ-Prolapse-%28July-2011%29.pdf.

[27] Glazener C.M., Breeman S., Elders A., Hemming C., Cooper K.G., Freeman R.M., Smith A.R., Reid F., Hagen S., Montgomery I. (2017). Mesh, graft, or standard repair for women having primary transvaginal anterior or posterior compartment prolapse surgery: Two parallel-group, multicentre, randomised, controlled trials (PROSPECT). Lancet.

[28] Haylen B.T., de Ridder D., Freeman R.M., Swift S.E., Berghmans B., Lee J., Monga A., Petri E., Rizk D.E., Sand P.K. (2010). An International Urogynecological Association (IUGA)/International Continence Society (ICS) joint report on the terminology for female pelvic floor dysfunction. Int. Urogynecol. J..

[29] Dietz H.P. (2012). Mesh in prolapse surgery: An imaging perspective. Ultrasound Obstet. Gynecol..

[30] Taithongchai A., Sultan A.H., Wieczorek P.A., Thakar R. (2019). Clinical application of 2D and 3D pelvic floor ultrasound of mid-urethral slings and vaginal wall mesh. Int. Urogynecol. J..

[31] Trutnovsky G., Nagele E., Ulrich D., Aigmüller T., Dörfler D., Geiss I., Reinstadler E., Angleitner-Flotzinger J., Ries J.J., Bjelic-Radisic V. (2016). German translation and validation of the Pelvic Organ Prolapse/Incontinence Sexual Questionnaire-IUGA revised (PISQ-IR). Int. Urogynecol. J..

[32] Christmann-Schmid C., Koerting I., Ruess E., Faehnle I., Krebs J. (2018). Functional outcome after laparoscopic nerve-sparing sacrocolpopexy: A prospective cohort study. Acta Obstet. Gynecol. Scand..

[33] Dua A., Radley S., Brown S., Jha S., Jones G. (2012). The effect of posterior colporrhaphy on anorectal function. Int. Urogynecol. J..

[34] Kudish B.I., Iglesia C.B. (2010). Posterior wall prolapse and repair. Clin. Obstet. Gynecol..

[35] Bedford N.D., Seman E.I., O’Shea R.T., Keirse M.J. (2015). Long-term outcomes of laparoscopic repair of cystocoele. Aust. N. Z. J. Obstet. Gynaecol..

[36] Chinthakanan O., Miklos J.R., Moore R.D. (2015). Laparoscopic Paravaginal Defect Repair: Surgical Technique and a Literature Review. Surg. Technol. Int..

[37] Maher C., Baessler K. (2006). Surgical management of anterior vaginal wall prolapse: An evidencebased literature review. Int. Urogynecol. J. Pelvic Floor Dysfunct..

[38] de Castro E.B., Brito L.G.O., Juliato C.R.T. (2020). Vaginal hysterectomy with bilateral sacrospinous fixation plus an anterior mesh versus abdominal sacrocervicopexy for the treatment of primary apical prolapse in postmenopausal women: A randomized controlled study. Int. Urogynecol. J..

[39] Lucot J.P., Cosson M., Bader G., Debodinance P., Akladios C., Salet-Lizée D., Delporte P., Savary D., Ferry P., Deffieux X. (2018). Safety of Vaginal Mesh Surgery Versus Laparoscopic Mesh Sacropexy for Cystocele Repair: Results of the Prosthetic Pelvic Floor Repair Randomized Controlled Trial. Eur. Urol..

[40] Najib B., Feghali I., Deval B. (2022). Laparoscopic Pectopexy for Recurrent Pelvic Organ Prolapse after Laparoscopic Sacrocolpopexy. J. Minim. Invasive Gynecol..

